# Supplementary material for: miR-582-5p Is a Tumor Suppressor microRNA Targeting the Hippo-YAP/TAZ Signaling Pathway in Non-Small Cell Lung Cancer
Source: Cancers (Basel). 2021 Feb 11;13(4):756. doi: 10.3390/cancers13040756 (PMC7918774; doi:10.3390/cancers13040756)
Supplement: Supplementary file 1 [file cancers-13-00756-s001.zip › cancers-1080336-supplementary materials/cancers-1080336 - layout-supplementary.docx]

Supplementary Materials

miR-582-5p is a Tumor Suppressor microRNA Targeting the Hippo-YAP/TAZ Signaling Pathway in Non-Small Cell Lung Cancer

Bowen Zhu, Mitheera V, Megan Finch-Edmondson, Yaelim Lee, Yue Wan, Marius Sudol and Ramanuj DasGupta


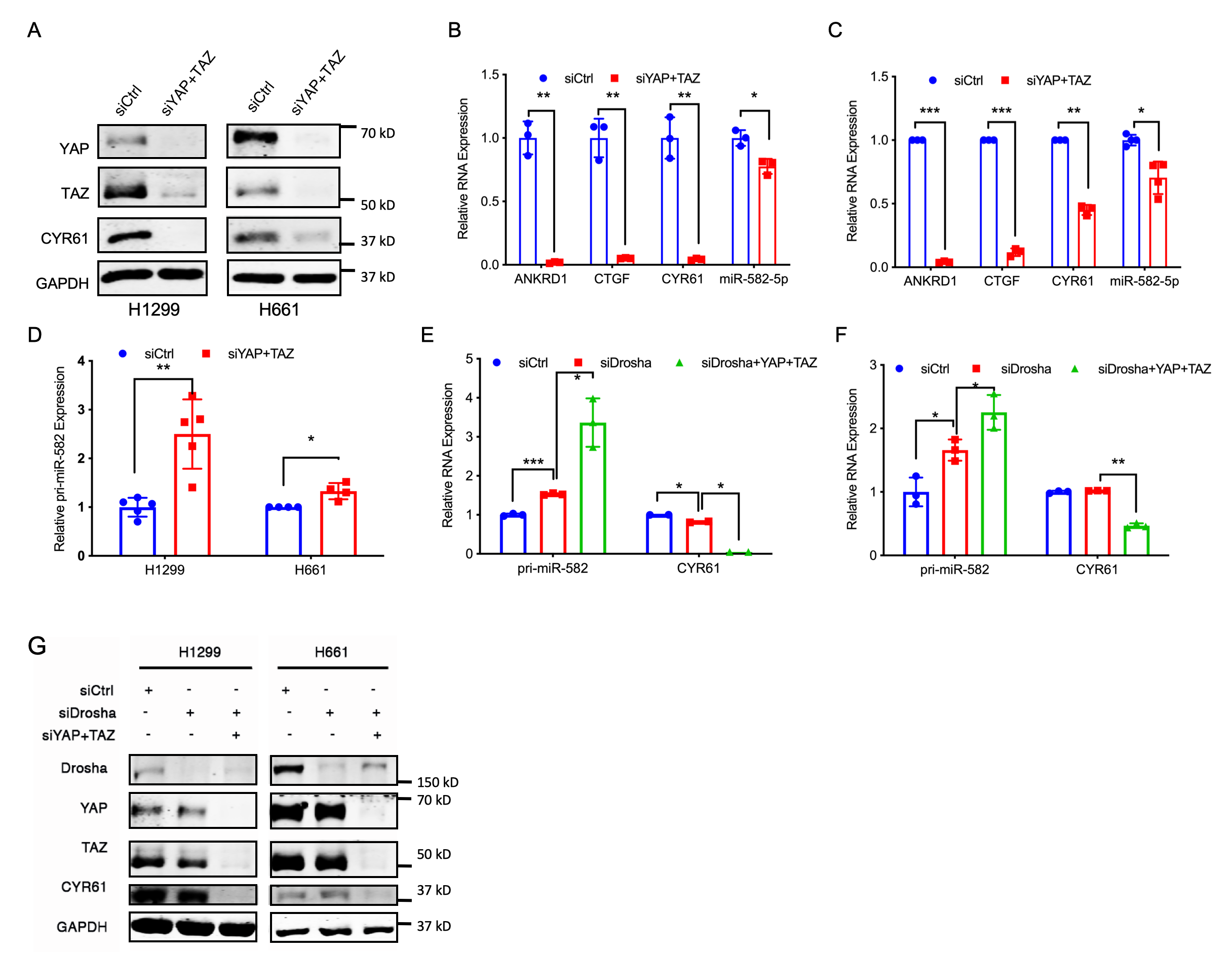


**Figure S1.** YAP/TAZ is required for miR-582-5p expression in NSCLC cells. (**A**) Western blotting was performed to confirm the knockdown of YAP and TAZ with siRNAs. GAPDH was used as a loading control. (**B**,**C**) Relative mRNA levels of YAP/TAZ-target genes, ANKRD1, CTGF, and CYR61, were evaluated alongside miR-582-5p expression in H1299 (**B**) and H661 (**C**) cells using RT-qPCR upon siRNA-mediated YAP/TAZ knockdown. (**D**) RT-qPCR was performed to evaluate pri-miR-582 levels in H1299 and H661 cells post-YAP/TAZ knockdown. (**E**,**F**) Relative mRNA levels of pri-miR-582 and CYR61 were determined upon siDrosha and siDrosha + siYAP + siTAZ treatments in H1299 (**E**) and H661 (**F**) cells. (**G**) Relative protein expression of YAP, TAZ, Drosha and CYR61 were determined with western blots in H1299 and H661 cells. Results are presented as the mean of 3–5 replicates as indicated with error bars representing standard deviation. Statistical analysis was conducted using Student’s t-test with * *p* < 0.05, ** *p* < 0.01, *** *p* < 0.001.


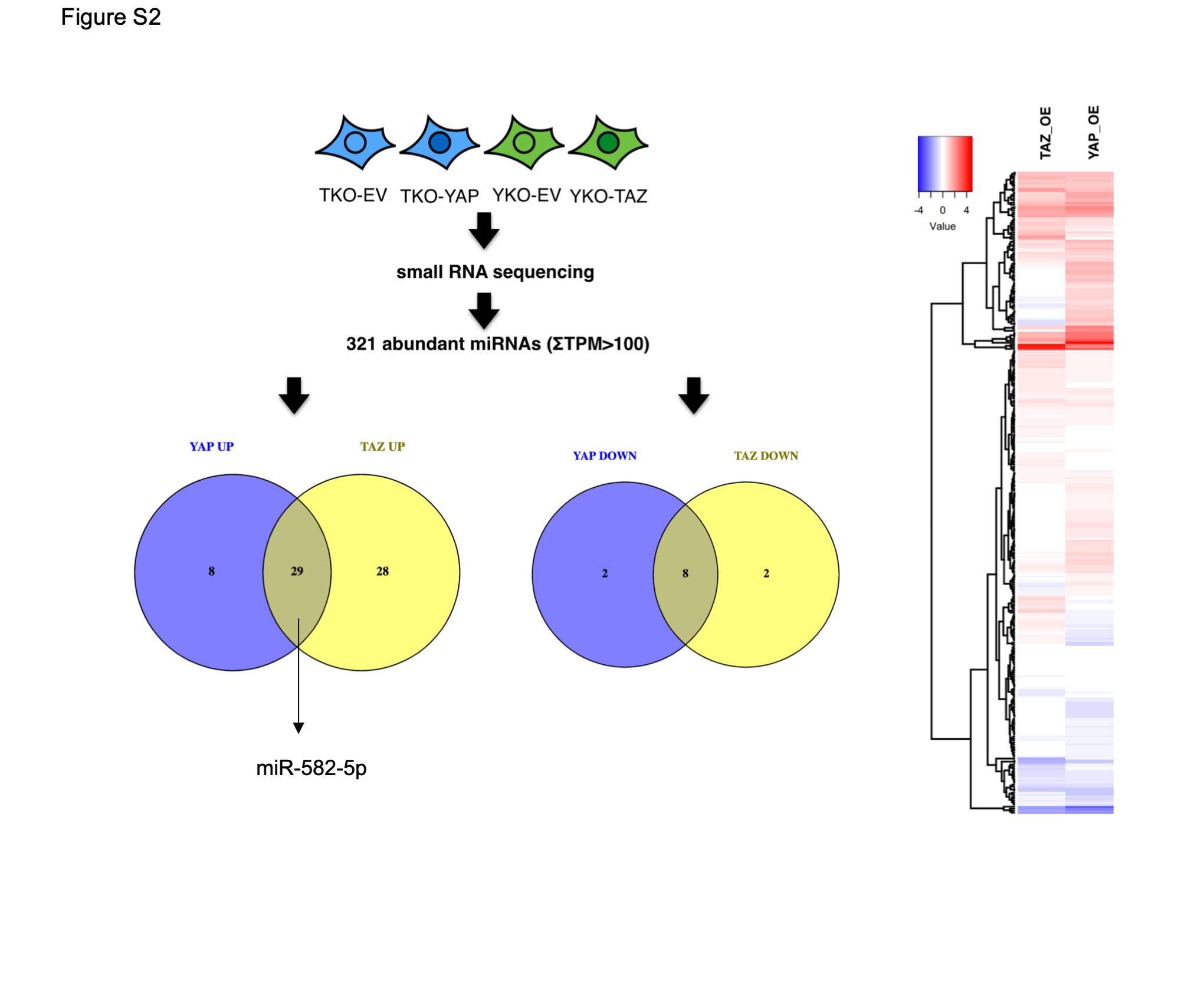


**Figure S2.** miR-582-5p is one of the microRNAs induced by YAP/TAZ overexpression. An illustration of the methodology employed in identifying miR-582-5p as a YAP/TAZ-induced miRNA from published small RNA sequencing data. In this dataset (35), small RNA sequencing (*n* = 2) was performed on (1–4) types of genetically modified MKN28 cells: Cells engineered with a TAZ-knockout (TKO) and subsequently transduced with either an empty vector (TKO-EV) (1) or an active form of YAP (TKO-YAP) (2), and cells engineered with a YAP-knockout to express empty vector (YKO-EV) (3) or active form of TAZ (YKO-TAZ) (4). A total of 321 miRNAs were identified to possess an aggregated TPM value greater than 100 in all eight libraries, terming these miRNAs as abundant miRNAs. Amongst the abundant miRNAs, 29 miRNAs were upregulated by both YAP and TAZ with fold changes greater than 4 (FC > 4) while 8 miRNAs were downregulated by both YAP and TAZ with fold changes greater than 2 (FC > 2). The Venn diagrams in this illustration reveal the dependency of abundant microRNAs on either YAP or TAZ or both YAP and TAZ. MiR-582-5p was identified as a YAP/TAZ-driven microRNA.


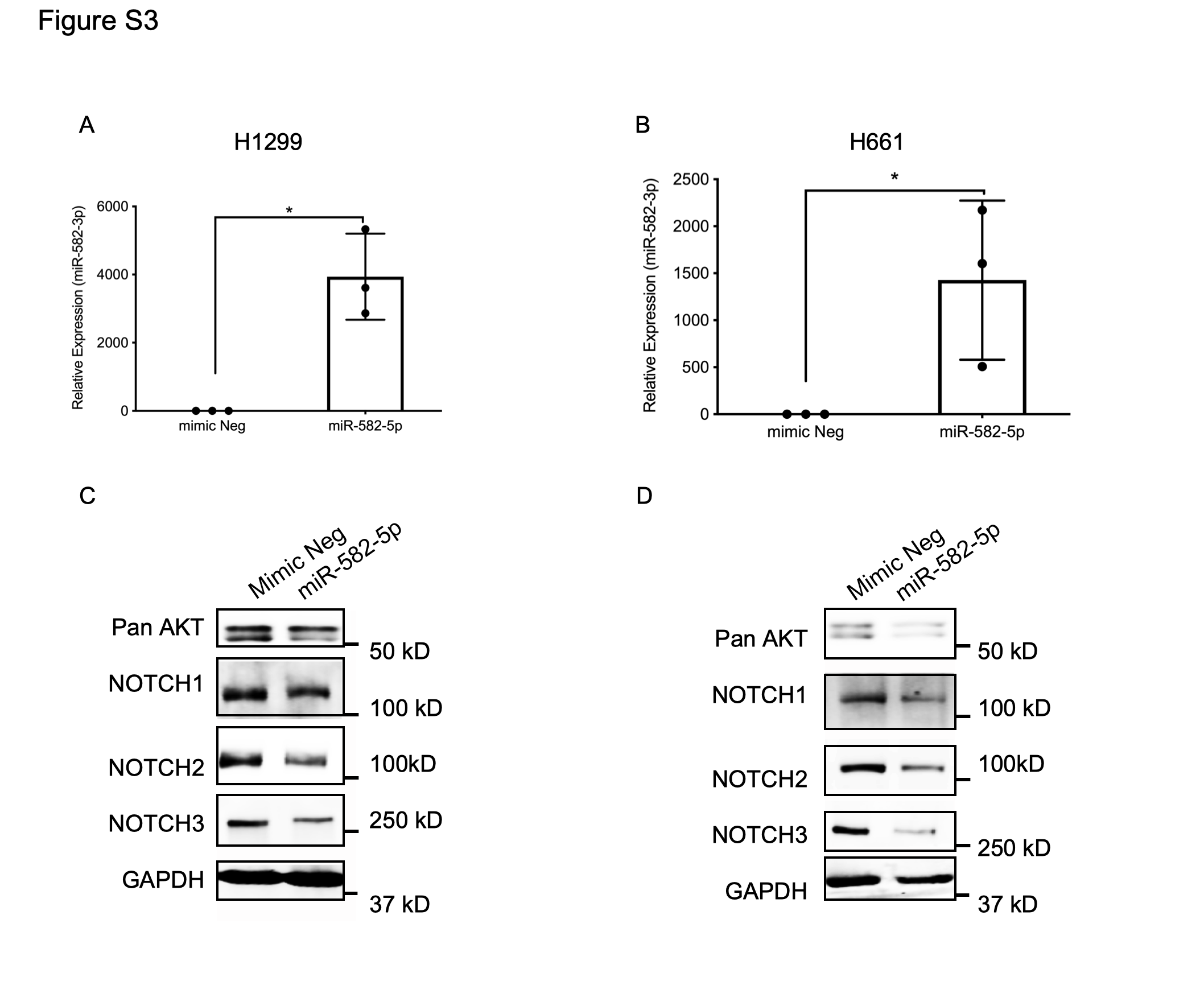


**Figure S3.** miR-582-5p overexpression leads to reductions of Notch and AKT proteins. (**A**,**B**) The overexpression of miR-582-5p was confirmed through microRNA RT-qPCR analysis in (A) H1299 and (**B**) H661 cell lines post-transfection with miR-582-5p mimics. (**C**,**D**) Western blot was conducted to evaluate the relative protein levels of pan-AKT and NOTCH1-3 following the transfection of miR-582-5p mimics in H1299 (**C**) and H661 (**D**) cells. GAPDH served as a loading control. Results are expressed as the mean of triplicate experiments with error bars representing standard deviation. Statistical analysis was conducted using Student’s t-test with * *p* < 0.05


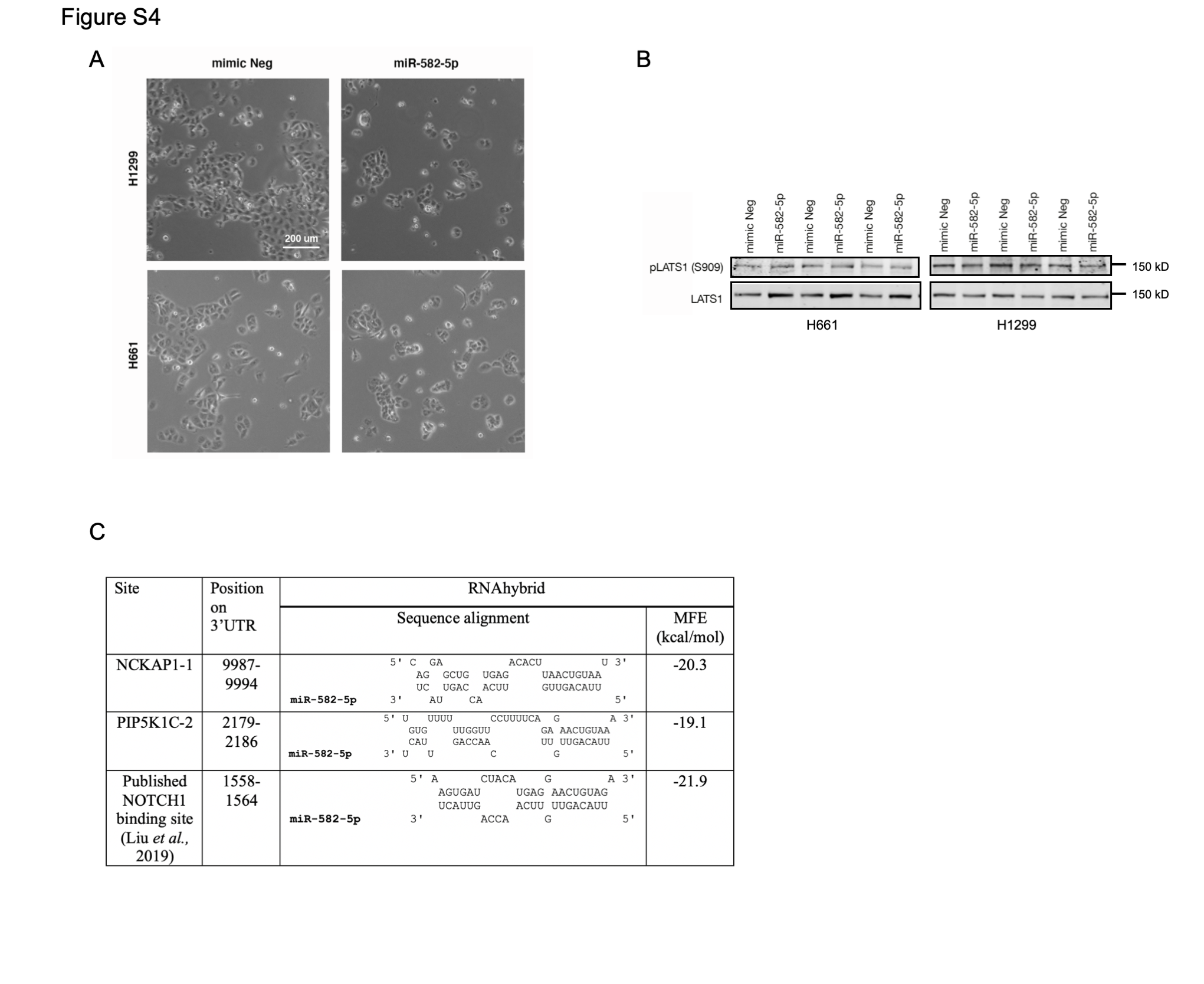


**Figure S4.** Effect of miR-582-5p overexpression on cell morphology and LATS phosphorylation. (**A**) Representative phase contrast images of increased cell rounding morphologies evident in H1299 and H661 cells upon overexpression of miR-582-5p in contrast to mimic-negative transfection. (**B**) Western blotting was conducted to determine the relative expression of phosphorylated LATS1 (S909) proteins upon miR-582-5p overexpression. Total LATS1 was used as an internal control.


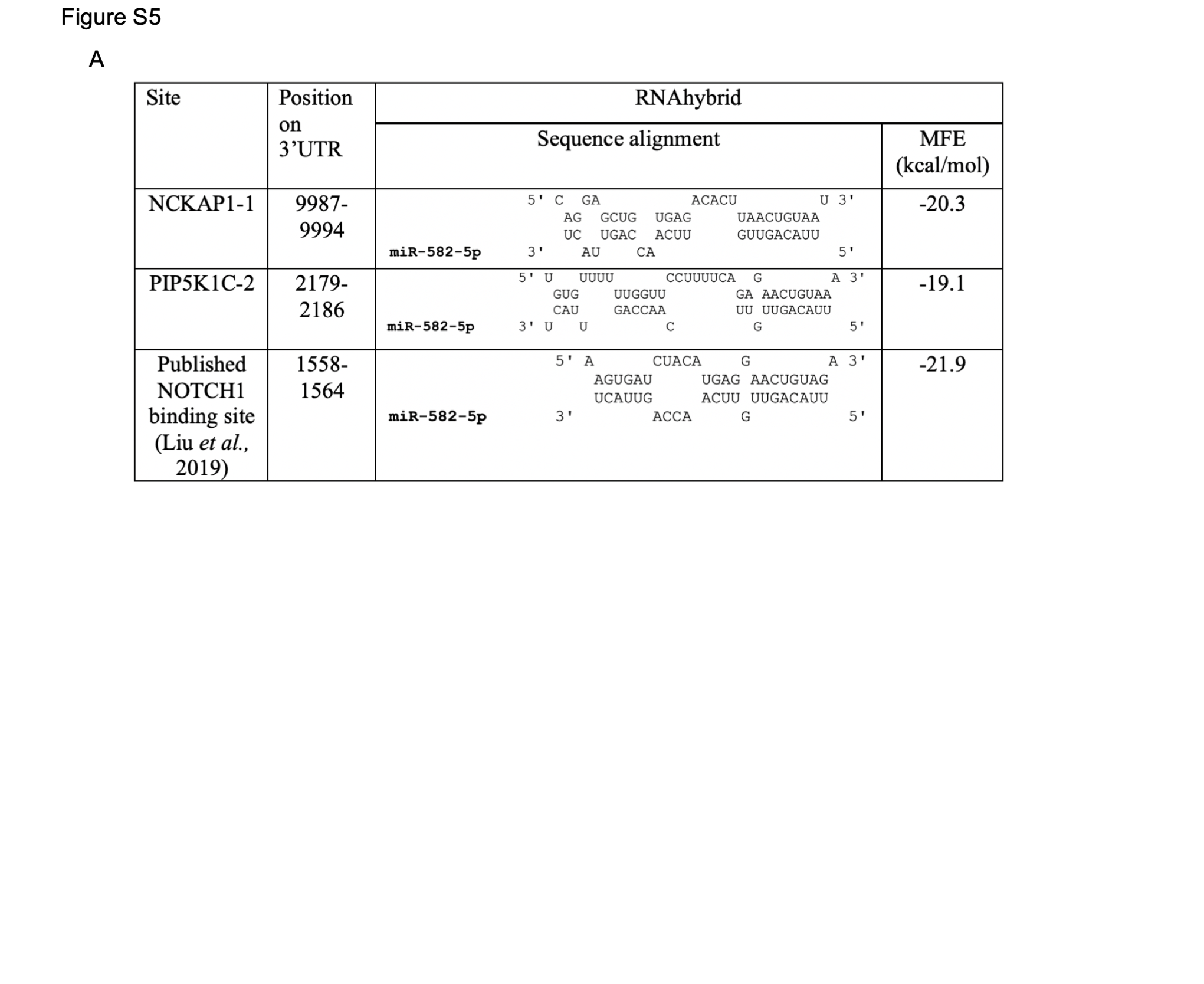


**Figure S5.** RNAhybrid analysis was carried out to examine the binding strength of miR-582-5p on NCKAP1-1 and PIP5K1C-2 sites, computed in minimum free energy (MFE), which was compared to that of the known miR-582-5p binding site on NOTCH1. G:U pairings were allowed in the alignment between miR-582-5p and the sites on NCKAP1, PIP5K1C, and NOTCH1 in order to compute the MFE of binding.


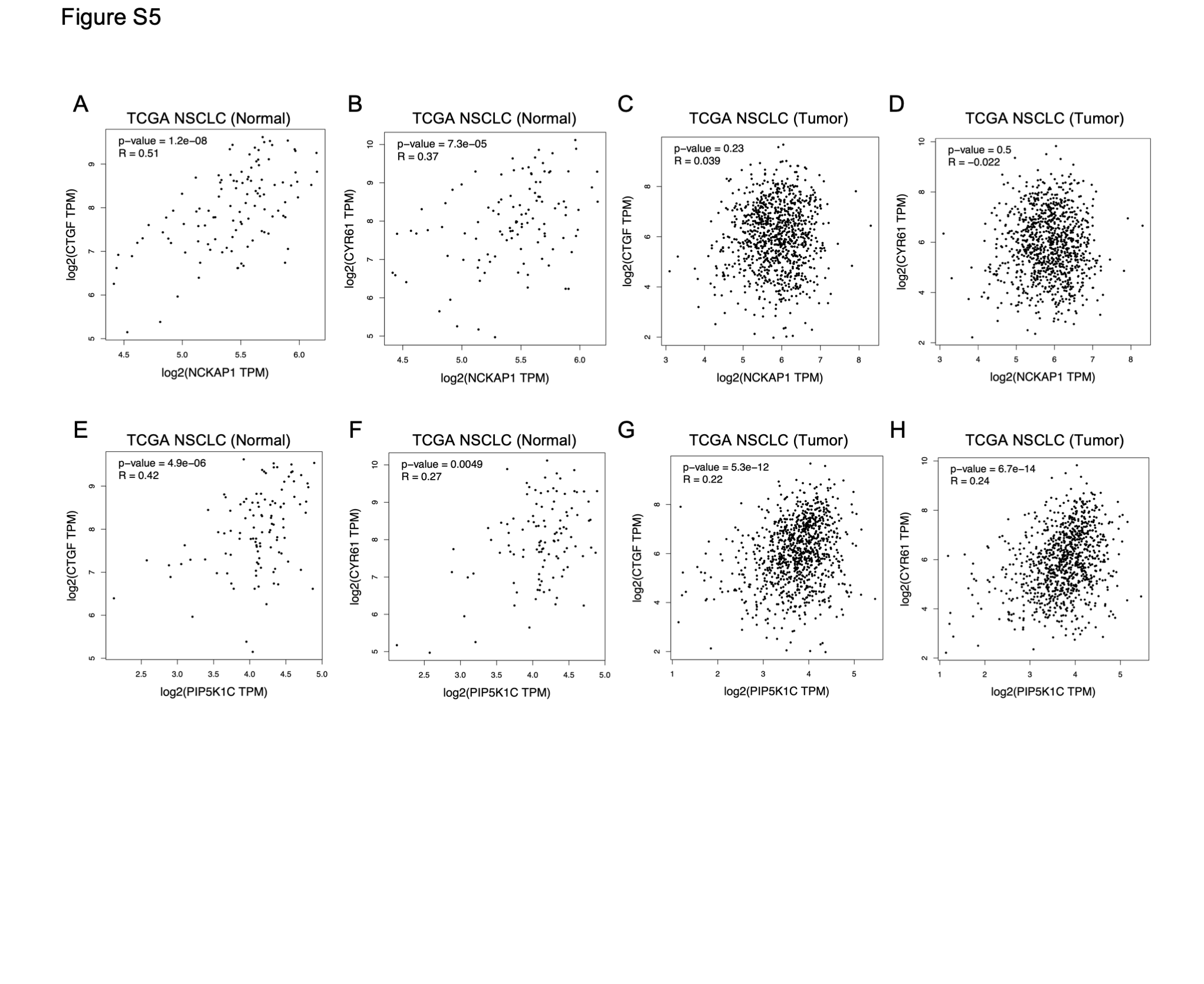


**Figure S6.** The expression of NCKAP1 and PIP5K1C positively correlates with that of YAP/TAZ transcriptional targets. Scatter plots obtained from The Cancer Genome Atlas (TCGA) illustrate the positive correlation in gene expression (Transcripts per million) between NCKAP1 or PIP5K1C and YAP/TAZ targets, namely, CYR61 and CTGF, in the tumor or normal cohorts of Non-Small Cell Lung Cancer (NSCLC) patients. TPM values were scaled using log2(TPM). R-values represent correlation coefficient and *p*-values reflect the statistical significance of the correlations, both of which were calculated using Pearson's correlation analysis.


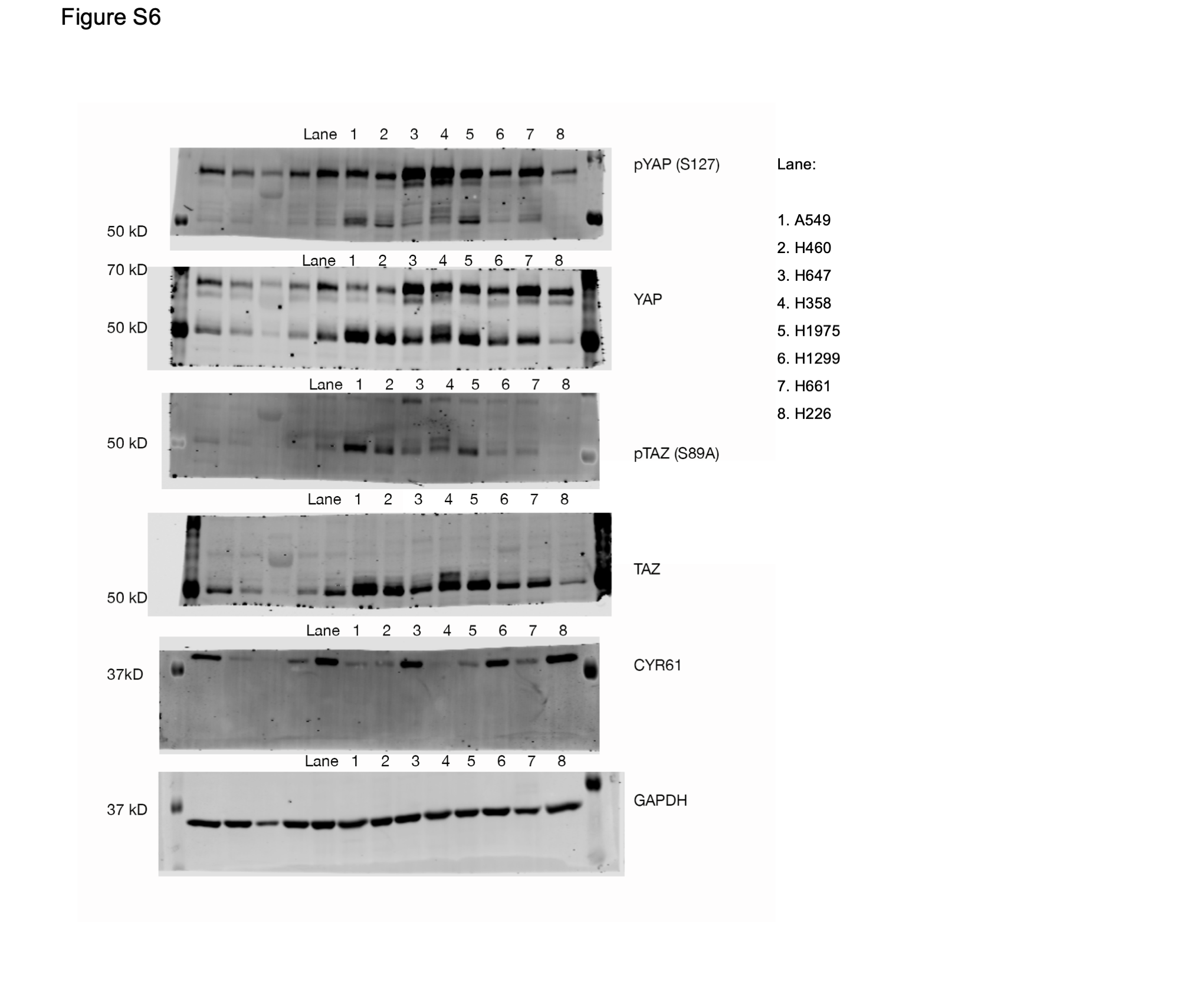


**Figure S7.** Raw Western blot data related to Figure 1.


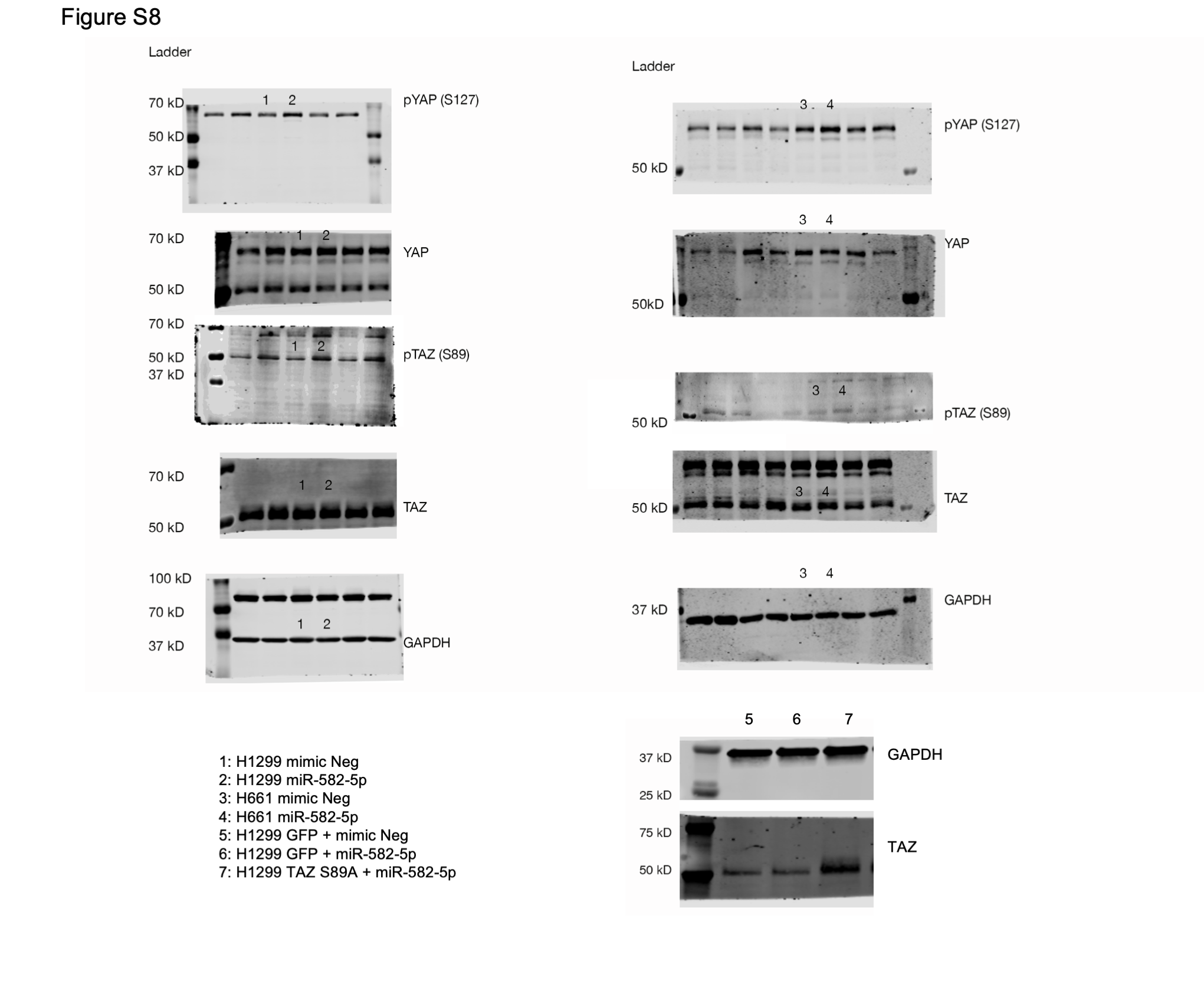


**Figure S8.** Raw Western blot data related to Figure 2.


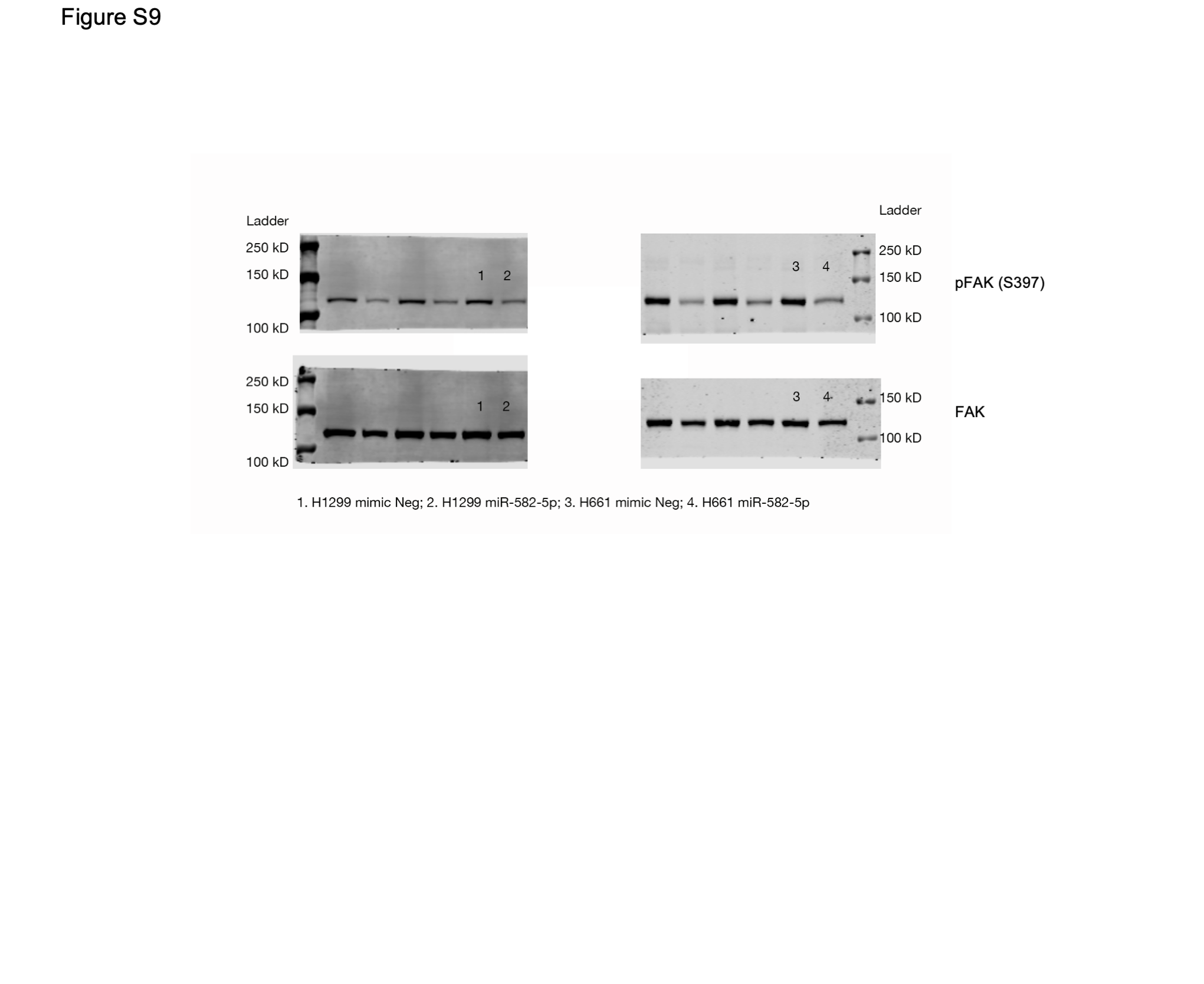


**Figure S9.** Raw Western blot data related to Figure 3.


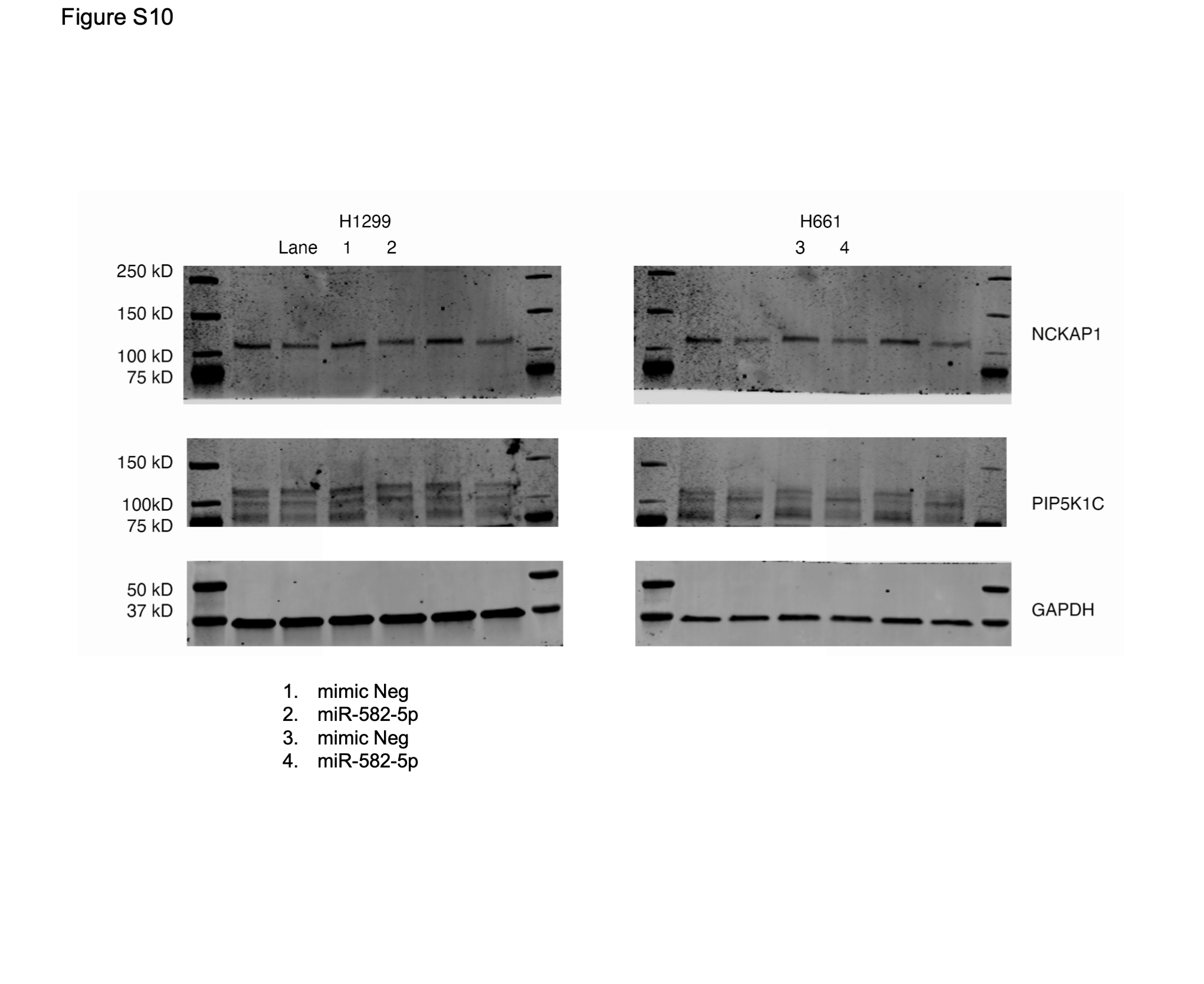


**Figure S10.** Raw Western blot data related to Figure 4.


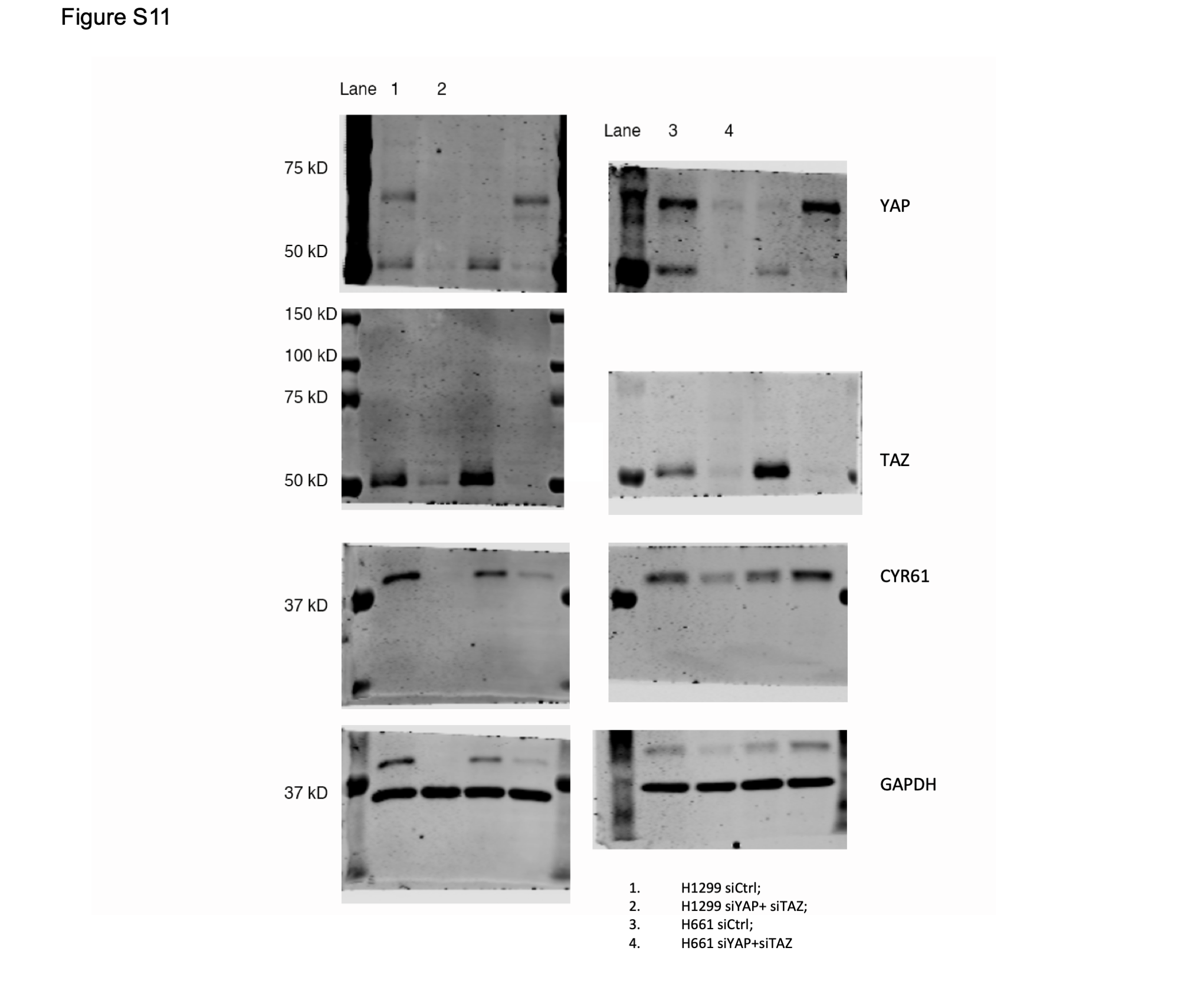


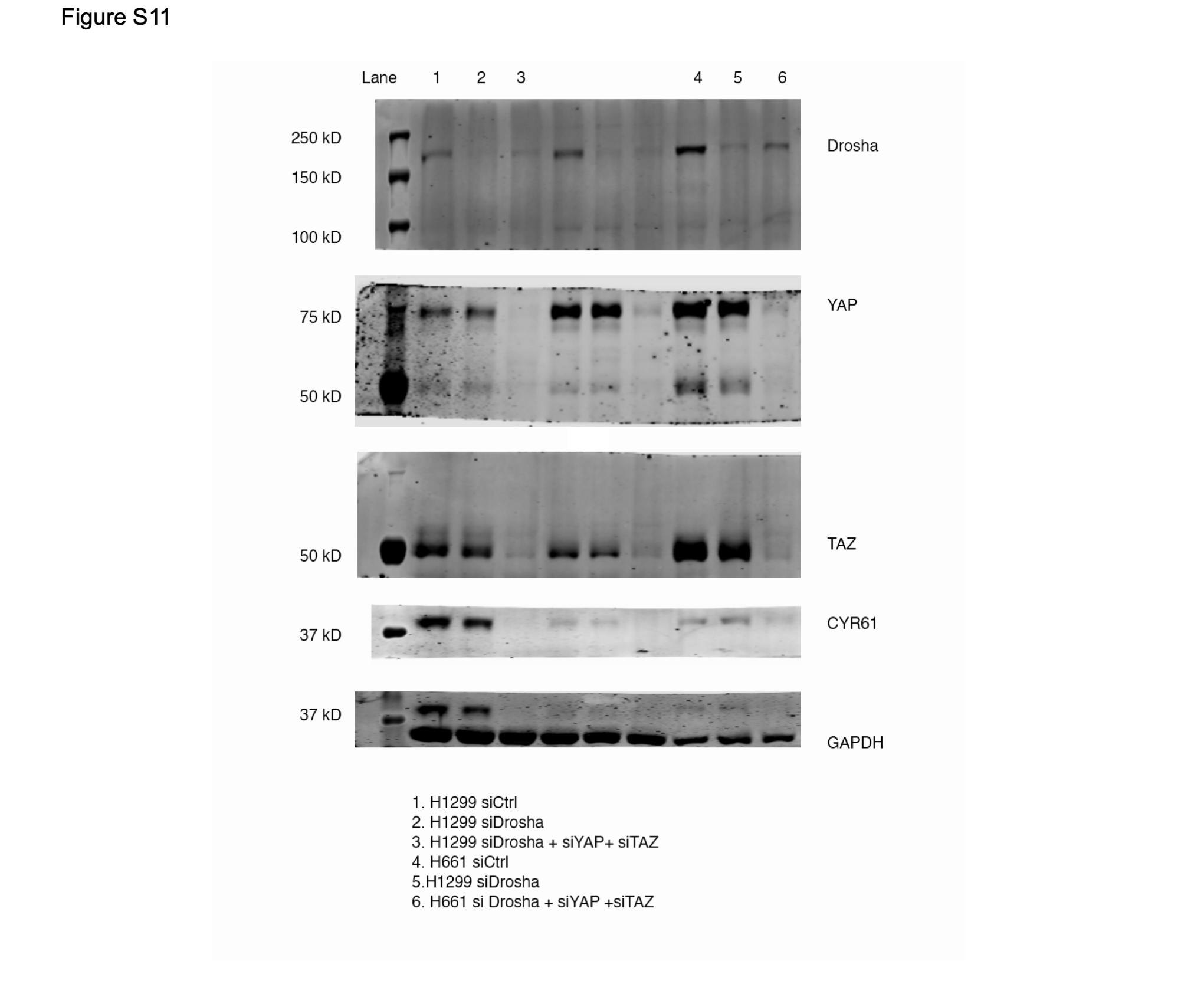


**Figure S11.** Raw Western blot data related to Figure S1.


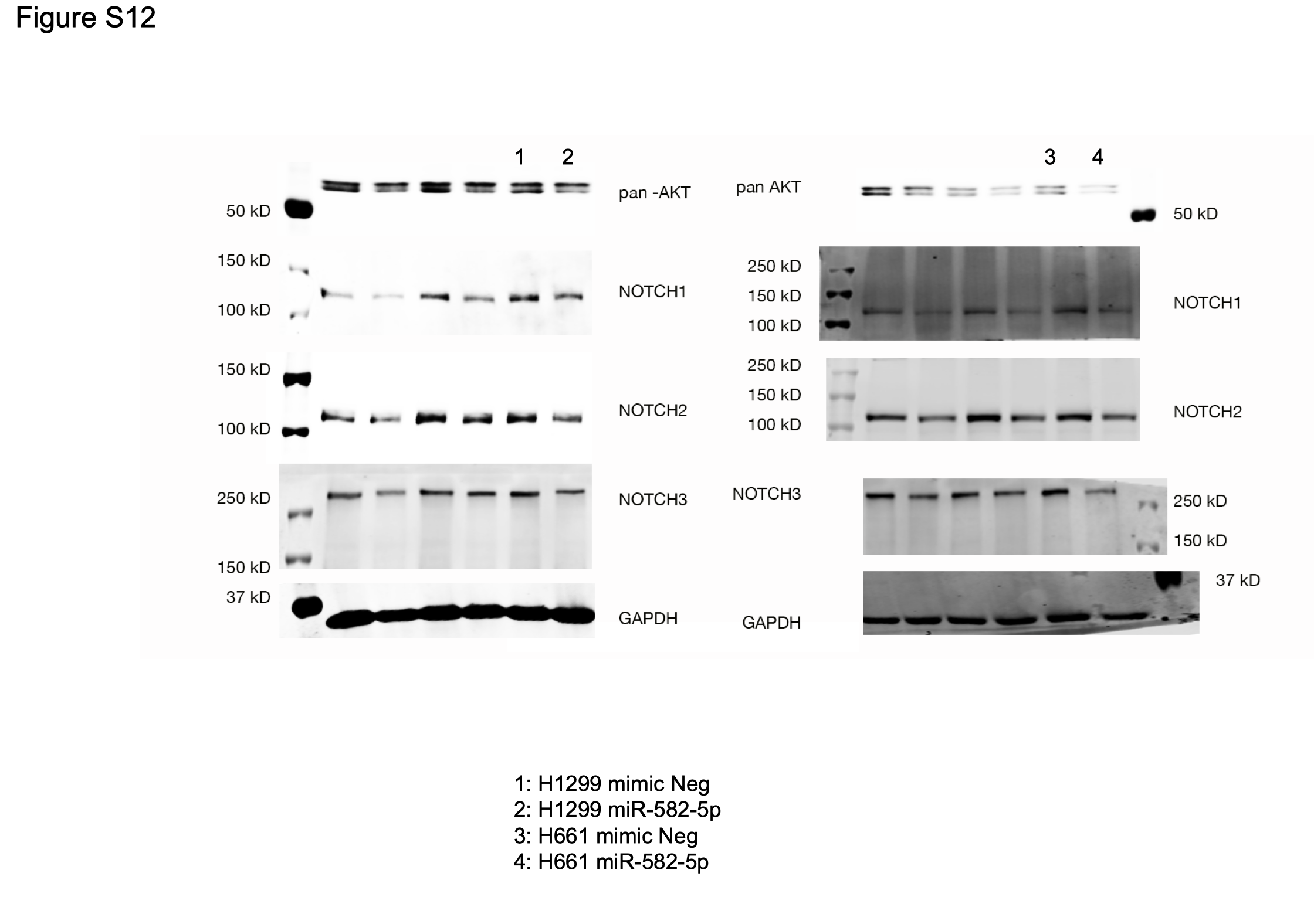


**Figure S12.** Raw Western blot data related to Figure S3.


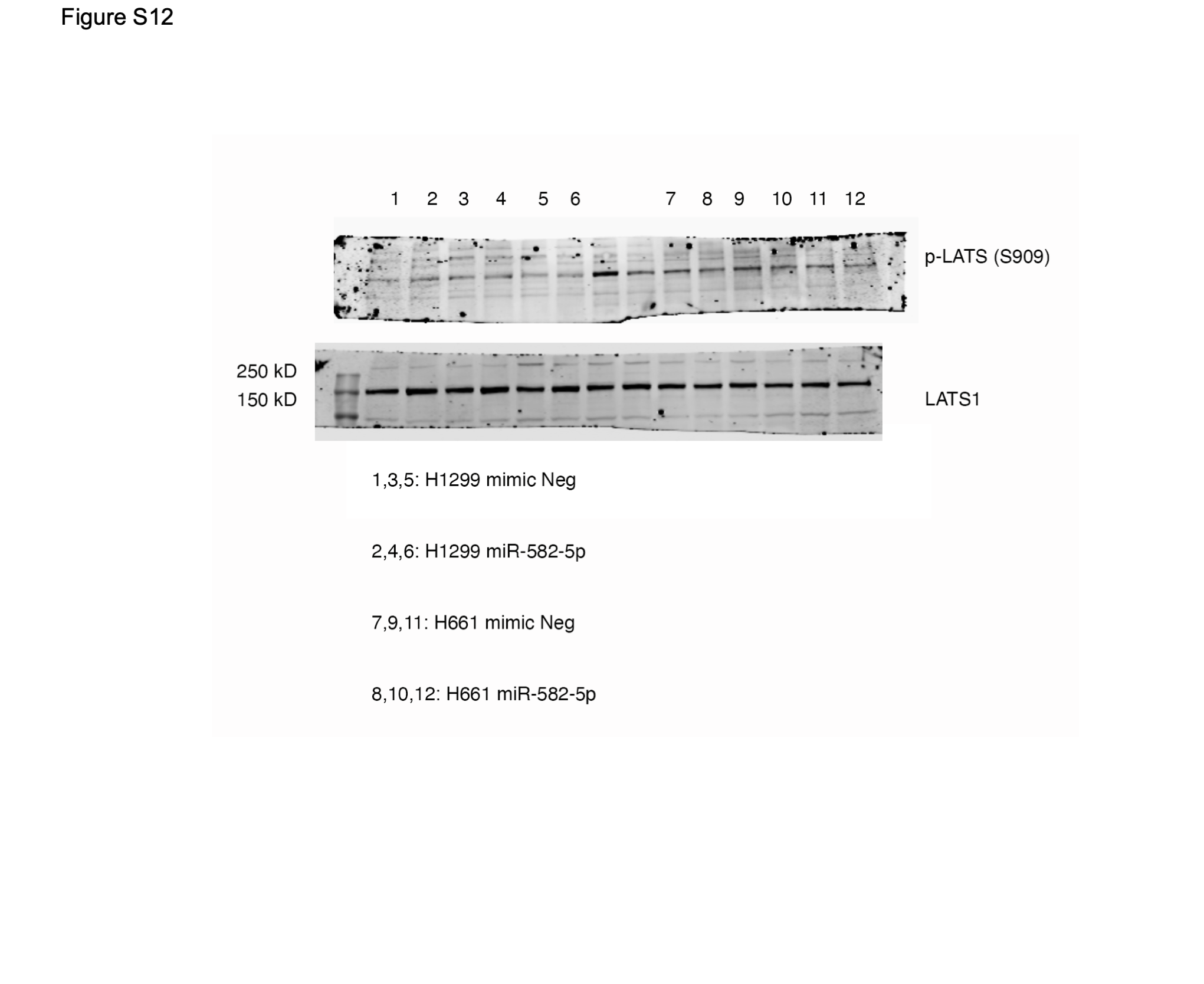


**Figure S13.** Raw Western blot data related to Figure S4.

**Table 1.** please view at the excel file.
